# Thermally activated delayed fluorescence (TADF) emitters: sensing and boosting spin-flipping by aggregation

**DOI:** 10.3762/bjoc.18.122

**Published:** 2022-09-08

**Authors:** Ashish Kumar Mazumdar, Gyana Prakash Nanda, Nisha Yadav, Upasana Deori, Upasha Acharyya, Bahadur Sk, Pachaiyappan Rajamalli

**Affiliations:** 1 Materials Research Centre, Indian Institute of Science, C. V. Raman Road, Bangalore, Karnataka, 560012, India

**Keywords:** intramolecular charge transfer, molecular aggregates, sensing, thermally activated delayed fluorescence (TADF)

## Abstract

Metal-free organic emitters with thermally activated delayed fluorescence (TADF) characteristics are emerging due to the potential applications in optoelectronic devices, time-resolved luminescence imaging, and solid-phase sensing. Herein, we synthesized two (4-bromobenzoyl)pyridine (BPy)-based donor–acceptor (D–A) compounds with varying donor size and strength: the emitter BPy-*p*TC with *tert*-butylcarbazole (TC) as the donor and BPy-*p*3C with bulky tricarbazole (3C) as the donor unit. Both BPy-*p*TC and BPy-*p*3C exhibited prominent emission with TADF properties in solution and in the solid phase. The stronger excited-state charge transfer was obtained for BPy-*p*3C due to the bulkier donor, leading to a more twisted D–A geometry than that of BPy-*p*TC. Hence, BPy-*p*3C exhibited aggregation-induced enhanced emission (AIEE) in a THF/water mixture. Interestingly, the singlet–triplet energy gap (Δ*E*_ST_) was reduced for both compounds in the aggregated state as compared to toluene solution. Consequently, a faster reverse intersystem crossing rate (*k*_RISC_) was obtained in the aggregated state, facilitating photon upconversion, leading to enhanced delayed fluorescence. Further, the lone-pair electrons of the pyridinyl nitrogen atom were found to be sensitive to acidic protons. Hence, the exposure to acid and base vapors using trifluoroacetic acid (TFA) and triethylamine (TEA) led to solid-phase fluorescence switching with fatigue resistance. The current study demonstrates the role of the donor strength and size in tuning Δ*E*_ST_ in the aggregated state as well as the relevance for fluorescence-based acid–base sensing.

## Introduction

Metal-free organic solid-state emitters have gained increasing interest in recent years due to the potential applications in optoelectronic devices [1–4], solid-phase sensing [5], bioimaging [6–7], security [8], and storage devices [9]. The optical and electrochemical properties of the organic emitters can be tuned by external stimuli, such as mechanical force, heat, solvent, acid and base fumes, etc. [10–11]. However, the fluorescence quantum yield of such emitters in the solid state is relatively low due to the aggregation-caused quenching (ACQ) effect [12–14] and limits practical applications. Hence, metal-free emitters with high photoluminescence quantum yield (PLQY) in solution and in the solid state and with multicolor tunability by external stimuli are crucial for task-specific applications.

Recently, donor–acceptor (D–A)-based organic emitters have shown tunable optical and electrochemical properties due to alteration of the intermolecular charge-transfer (ICT) interactions [15]. Several molecular designs were proposed to adjust the ICT interactions through covalent D–A linking. The separation of the highest occupied molecular orbital (HOMO) and the lowest unoccupied molecular orbital (LUMO) of the donor and acceptor molecules leads to a reduction of the singlet–triplet energy gap (Δ*E*_ST_) [16–17]. The low Δ*E*_ST_ facilitates the exciton upconversion through reverse intersystem crossing (RISC) [2], which is commonly known as thermally activated delayed fluorescence (TADF) [16]. Theoretically, this has up to 100% internal quantum efficiency and could replace heavy metal-based emitters in organic light-emitting diode (OLED) devices [18]. Nevertheless, many TADF emitters suffer from quenching of the emission due to the aggregation-caused quenching effect [19–20]. The strong π–π-stacking interactions in the solid or aggregated state may lead to emission quenching. Therefore, an appropriate molecular design to suppress π–π stacking should help to obtain a strong emission in solution as well as in the solid state.

Solid-state organic emitters with reversible fluorescence switching are emerging for the sensing of pollutant acid vapor [21–23]. However, quickly detecting organic acid vapor at ambient conditions is challenging for solid-state detectors. Therefore, developing a solid-state emitter with reversible switching of the optical properties by external stimuli, such as acid and base vapors, is important. As per the recent literature, such sensors are being developed mainly by focusing on photophysical processes, such as photoinduced electron transfer (PET), excited-state intramolecular proton transfer (ESIPT), fluorescence resonance energy transfer (FRET), etc. [21–22,24–25]. Several literature reports have also demonstrated the switching of fluorescence in designing heteroatom-containing (N, O, S, etc.) chromophores [26]. The lone-pair electrons of heteroatoms can possess a certain degree of basicity, which results in sensitivity to acid. Consequently, the lone pair may be protonated and deprotonated by consecutively adding acid and base. The disruption of the molecular conjugation or ICT interactions upon protonation or deprotonation would lead to switching of the optical properties. While there are many reports on acid–based sensors, TADF emitter-based sensors are rare in the literature.

In this context, we chose the D–A molecular design to demonstrate the solid-phase acid–base sensing process and to determine the RISC rate (*k*_RISC_). The selection of different donors with varying donor strength and size should help to understand the charge transfer (CT) interactions and D–A twist. Controlling the D–A twisting by varying the donor unit should be beneficial for suppressing nonradiative deactivation channels, leading to aggregated- or solid-state emission properties. To this end, we chose *tert*-butylcarbazole (TC) and bulky tricarbazole (3C), respectively, as donor unit in combination with a (4-bromobenzoyl)pyridine (BPy) acceptor core to demonstrate Δ*E*_ST_ tuning in solution and in an aggregated state, including the aggregation-induced enhanced emission (AIEE) characteristics. Further, solid-phase fluorescence switching by alternatingly adding acid and base vapors demonstrated the sensing ability. Therefore, the current study deciphers the role of molecular design and donor size in tuning Δ*E*_ST_ in an aggregated state and in fluorescence switching by acid–base addition.

## Results and Discussion

### Design and synthesis

**Scheme 1:**
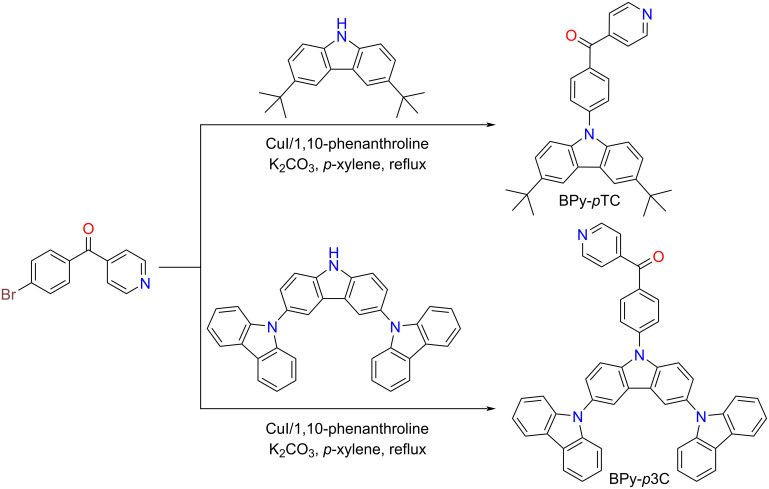
Synthetic schemes of BPy-*p*TC and BPy-*p*3C.

Several BPy derivatives have been reported to lower Δ*E*_ST_ in D–A pairs to promote spin upconversion from the low-lying triplet excited state to the singlet state via RISC [17,27]. Heteroatom lone-pair electrons are sensitive to acid and, once protonated, to base, which would facilitate the tuning of optical properties in such media [21–22]. Herein, (4-(3,6-di-*tert*-butyl-9*H*-carbazol-9-yl)phenyl)(pyridin-4-yl)methanone (BPy-*p*TC) and (4-(9'*H*-[9,3':6',9''-tercarbazol]-9'-yl)phenyl)(pyridin-4-yl)methanone (BPy-*p*3C) were synthesized via Ullmann coupling following the reported protocol [28]. These two TADF emitters have already been synthesized and device fabrication had been demonstrated. However, detailed photophysical investigations in the aggregated state have not yet been performed [28]. The acceptor core BPy, coupled with carbazole derivatives, produced BPy-*p*TC and BPy-*p*3C, respectively (Scheme 1). The detailed synthetic procedures and characterization data are given in the Experimental section and in Schemes S1 and S2 in Supporting Information File 1. Both compounds were further purified by temperature-gradient vacuum sublimation and characterized by ^1^H and ^13^C NMR spectroscopy (Figures S9–S12, Supporting Information File 1) as well as high-resolution mass spectrometry.

### Photophysical studies and DFT calculations

**Figure 1:**
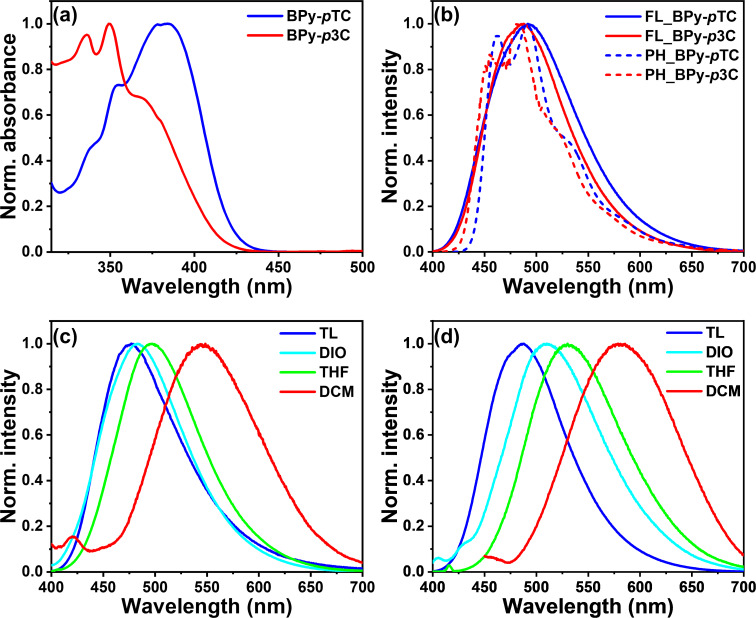
(a) Normalized absorption spectra of BPy-*p*TC and BPy-*p*3C in toluene at room temperature; (b) normalized fluorescence (λ_ex_ = 375 nm, 10 µM in toluene) spectra at ambient temperature and phosphorescence spectra (λ_ex_ = 375 nm, 10 µM) of BPy-*p*TC and BPy-*p*3C at 77 K; emission spectra (λ_ex_ = 375 nm) of BPy-*p*TC (c) and BPy-*p*3C (d) in various polar solvents. (TL: toluene, DIO: 1,4-dioxane, THF: tetrahydrofuran, DCM: dichloromethane).

The UV–vis absorption and emission spectra of BPy-*p*TC and BPy-*p*3C were recorded in solvents with varying polarity (Figure 1 and Table 1). BPy-*p*TC exhibited prominent absorption bands at λ_abs_ = 337, 355, and 383 nm. The peaks at λ_abs_ = 337 and 335 nm were due to the π–π* and n–π* transitions [29], whereas the broad peak at λ_abs_ = 383 nm was due to the ICT transitions. Similarly, three peaks centered at λ_abs_ = 336, 350, and 368 nm were observed for BPy-*p*3C. The ICT band of BPy-*p*3C (λ_abs_ = 368 nm) was blue-shifted as compared to BPy-*p*TC (λ_abs_ = 383 nm) due to the weaker ground state CT interactions. As a result, a stronger molar extinction coefficient was obtained for the CT band in BPy-*p*TC (ε_373 nm_ = 17310 M^−1^⋅cm^−1^) as compared to BPy-*p*3C (ε_365 nm_ = 14690 M^−1^⋅cm^−1^).

**Table 1:** Photophysical properties of BPy-*p*TC and BPy-*p*3C.^a^

compound	λ_em_ (nm)	τ^PF^ (ns)	τ^DF^ (µs)	Φ_total_ (%)	Δ*E*_ST_ (eV)

BPy-*p*TC	476	6.5	0.5	19	0.121
BPy-*p3*C	488	5.1	0.4	26	0.047

^a^Measured in degassed toluene (10 µM).

Computational calculations were performed to understand the ground state electronic communication between the donor and acceptor in BPy-*p*TC and BPy-*p*3C using the Gaussian 16 program package (Figure S1 as well as Tables S1 and S2 in Supporting Information File 1). The LUMO of both compounds was predominantly located on the acceptor core, whereas the HOMO was distributed mainly over the donor unit for both compounds and extended to the adjacent phenyl π-spacer ring only for BPy-*p*TC (Figure S1, Supporting Information File 1). As a consequence, a higher oscillator strength (*f* = 0.213) was obtained for BPy-*p*TC as compared to BPy-*p*3C (*f* = 0.073, Table S1, Supporting Information File 1). Additionally, a 48° and 51° (59° for the second donor and overall D–A dihedral = 97°) D–A dihedral angle was obtained for BPy-*p*TC and BPy-*p*3C, respectively (Figure S1, Supporting Information File 1). Therefore, the coplanarity between the D–A linkage in BPy-*p*TC facilitated the prominent ground-state communication from the donor to the acceptor core, as compared to the twisted BPy-*p*3C (Figure S1, Supporting Information File 1) [30].

The steady-state emission of both BPy-*p*TC and BPy-*p*3C was recorded in different polar media (Figure 1c and Figure 1d). The BPy-*p*TC emission lay in the cyan region, and the peak was centered at λ_em_ = 476 nm in toluene (Figure 1b). The emission spectra of BPy-*p*TC were red-shifted in polar media, and the emission maxima changed from λ_em_ = 476 nm in toluene to λ_em_ = 497 nm in THF and to λ_em_ = 545 nm in DCM (Figure 1c). Similarly, blue emission was observed in toluene at λ_em_ = 488 nm for BPy-*p*3C, and it red-shifted to λ_em_ = 530 nm in THF and to λ_em_ = 582 nm in polar DCM (Figure 1d). As compared to the absorption spectra, the significant solvatochromic shifts in the fluorescence spectra suggested the presence of a highly dipolar excited state with a stronger ICT character, in contrast to the ground state of the molecule. But the Stokes shift of BPy-*p*3C (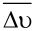 = 6640 cm^−1^ in toluene and 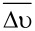 = 9991 cm^−1^ in DCM) was always higher than that of BPy-*p*TC (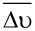 = 5145 cm^−1^ in toluene and 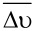 = 7761 cm^−1^ in DCM) in all polar media, indicating a highly dipolar excited state (Figure 1c and Figure 1d as well as Tables S3 and S4 in Supporting Information File 1). The large Stokes shift and spectral broadening indicated a highly polarized ICT state in both compounds (Figure 1c and Figure 1d as well as Tables S3 and S4 in Supporting Information File 1) [30]. The excited-state CT characteristics were further probed using the Lippert–Mataga plot for both the compounds (Figure S2 and Tables S3–S5 in Supporting Information File 1) [29]. A larger Stokes shift and transient dipole (µ_E_−µ_G_) than in BPy-*p*TC were observed in BPy-*p*3C (Table S5, Supporting Information File 1). This indicated the relatively stronger excited-state CT interactions in BPy-*p*3C (Figure 1c and Figure 1d as well as Tables S3–S5 in Supporting Information File 1).

The phosphorescence spectra of BPy-*p*TC and BPy-*p*3C were recorded in toluene at 77 K (Figure 1b). The structural phosphorescence bands were obtained for both compounds. The nature of the phosphorescence bands indicated the locally excited (^3^LE) character of the T_1_ state. The onset of the fluorescence spectrum (energy of S_1_) in toluene at room temperature and the onset of the phosphorescence spectrum (energy of T_1_) in toluene at 77 K was chosen to estimate the Δ*E*_ST_ values of the emitters (Figure 1b) [31]. A smaller singlet–triplet splitting was observed for BPy-*p*3C (Δ*E*_ST_ = 0.047 eV) as compared to BPy-*p*TC (Δ*E*_ST_ = 0.121 eV) due to the twisted molecular geometry (Figure 1 and Table 1). The small Δ*E*_ST_ values indicated a possible RISC process from the T_1_ to the S_1_ state for both the compounds. The photophysical properties of both the emitters in toluene are summarized in Table 1.

### Time-resolved spectroscopy

**Figure 2:**
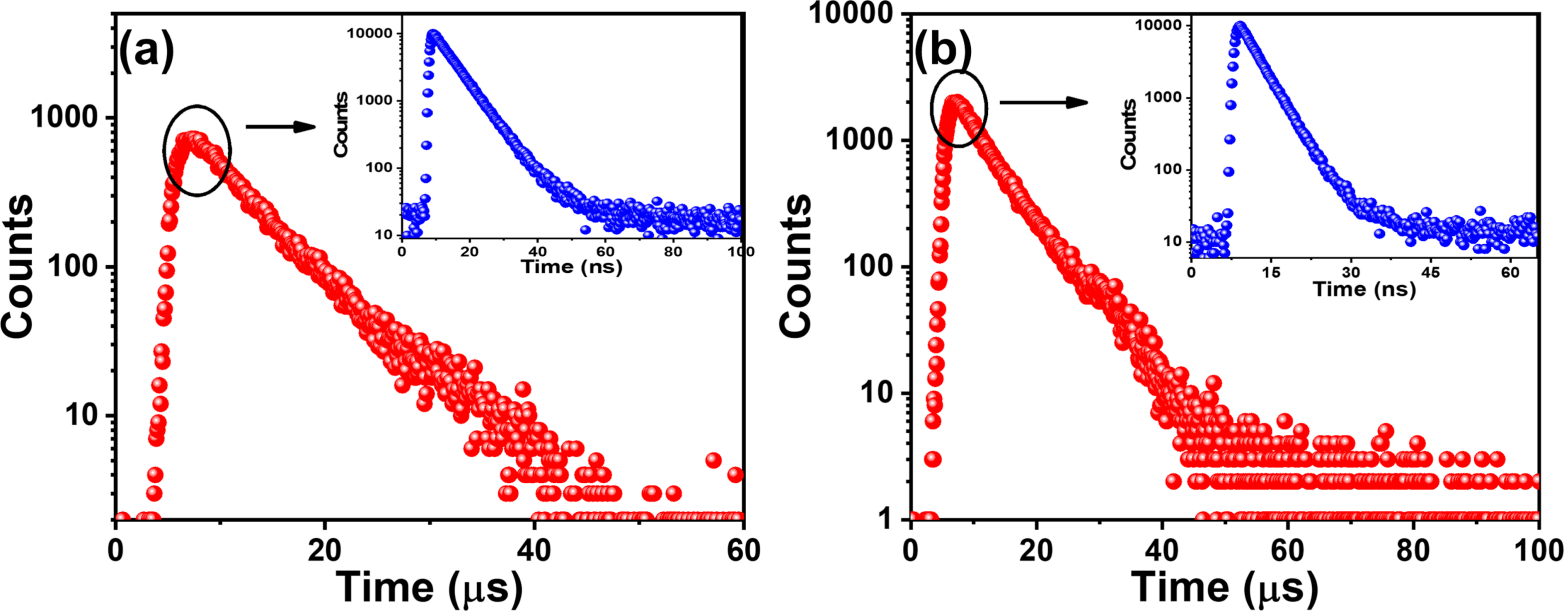
Transient photoluminescence decay (λ_ex_ = 375 nm) of (a) BPy-*p*TC and (b) BPy-*p*3C in degassed THF (10 µM) at room temperature.

Further, the fluorescence lifetimes on the nanosecond and microsecond timescales of both emitters were recorded in degassed THF to understand the photon upconversion process (Figure 2 and Tables S6 and S7 in Supporting Information File 1). Emission decay on the nanosecond and microsecond timescales was obtained for BPy-*p*TC, with decay lifetimes of 6.2 ns and 5.8 µs, respectively (Figure 2a as well as Table S6 in Supporting Information File 1). In comparison, a biexponential fluorescence decay with average lifetimes of τ_avg_ = 3.5 ns and 6.6 ns was obtained for BPy-*p*3C (Figure 2b as well as Table S7 in Supporting Information File 1). The short component was due to the prompt fluorescence (PF), and the long component was the delayed fluorescence (DF) [32]. Hence, both emitters displayed prominent DF due to the small Δ*E*_ST_, facilitating the photon upconversion from the T_1_ to the S_1_ state through an RISC process.

### Aggregation-induced enhanced emission (AIEE)

**Figure 3:**
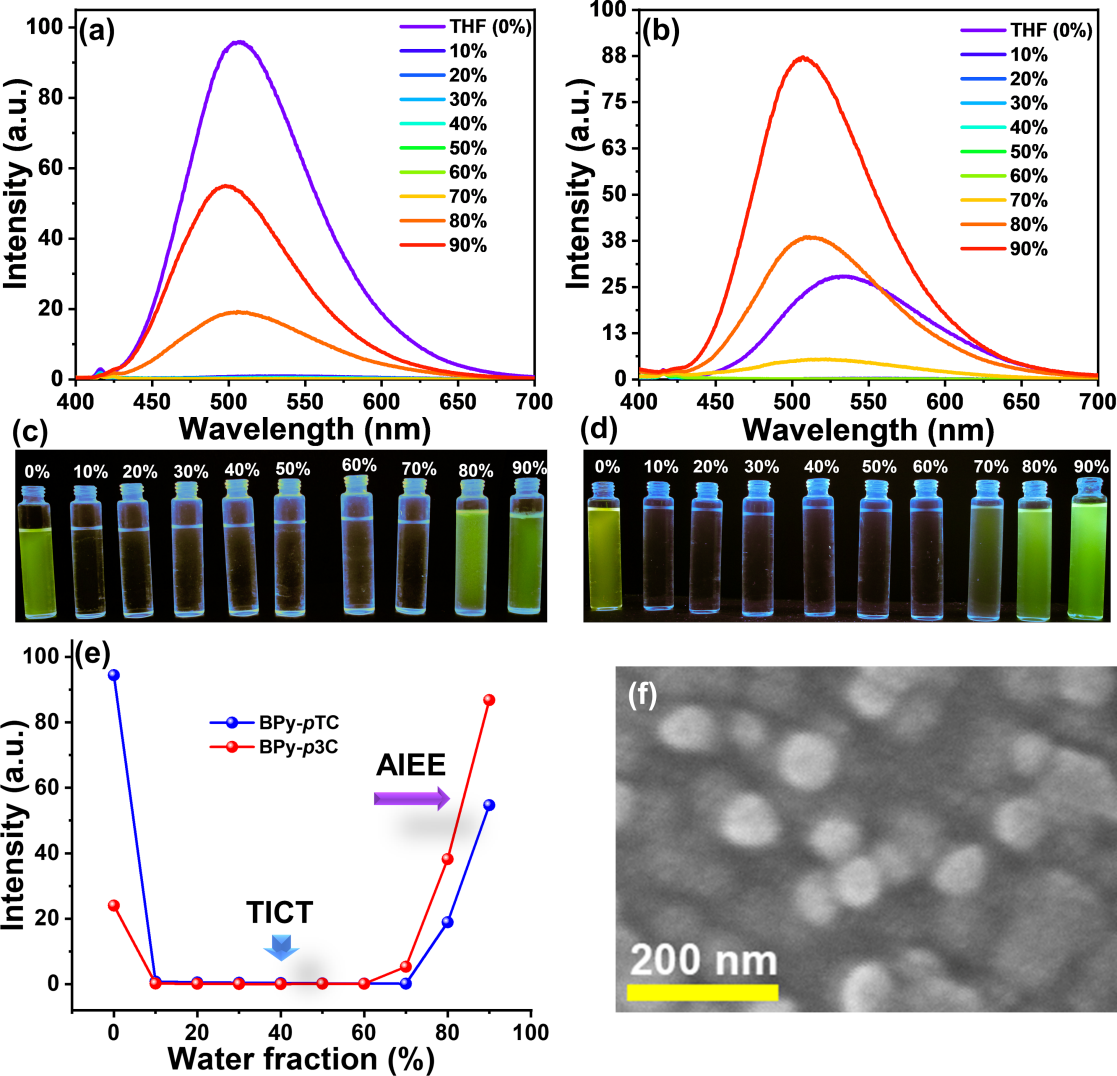
AIEE studies: Emission spectra (λ_ex_ = 375 nm, 10 µM) of (a) BPy-*p*TC and (d) BPy-*p*3C in THF with increasing water fraction (in vol %) at room temperature. Digital photographs of (c) BPy-*p*TC and (d) BPy-*p*3C in THF/water solutions under exposure to a UV lamp (λ_ex_ = 365 nm). (e) Plot of PL intensity vs water fraction of BPy-*p*TC and BPy-*p*3C depicting the AIEE phenomenon in BPy-*p*3C. (f) SEM image of BPy-*p*TC aggregates formed in a 90 vol % water/THF mixture.

In order to determine the molecular aggregate formation of BPy-*p*TC and BPy-*p*3C and the impact on the optical properties, we carried out aggregation studies in THF/water mixtures (Figure 3) [33]. BPy-*p*TC emits in the blue-green region at λ_em_ = 497 nm in THF with a PLQY of 12.7% (Figure 3a). The emission intensity was reduced to near 0 upon increasing the water fraction from 10 to 70 vol % in the THF solution of BPy-*p*TC (Figure 3a and Figure 3c). The formation of a dark twisted intramolecular CT state led to fluorescence quenching in the highly polar water/THF mixture (10–70 vol % water, Figure 3a, c, and e) [34–36]. It again started glowing when the water fraction was increased beyond 80 vol % (Figure 3a, c, and e). The formation of molecular aggregates in >80 vol % water/THF mixtures led to the regaining of fluorescence in BPy-*p*TC (Figure 3a, c, e, and f). A slight blue shift was also observed in 80–90 vol % water/THF mixtures of BPy-*p*TC due to restriction of D–A rotation in the aggregated state, which led to diminished CT [34]. The nanoaggregates (in 90 vol % water/THF) of BPy-*p*TC were characterized using scanning electron microscopy (SEM). The SEM image showed the spherical nanoaggregates of BPy-*p*TC, with 95 nm diameter (Figure 3f). The BPy-*p*3C compound emitted in the green region at λ_em_ = 530 nm in THF, with a PLQY of 3.6% (Figure 3a). Similar to BPy-*p*TC, for BPy-*p*3C, a dark state in 10–70 vol % water/THF mixtures and emissive aggregates in 80–90 vol % water/THF mixtures were obtained (Figure 3b, d, and e). Interestingly, AIEE was obtained only for BPy-*p*3C in a 90 vol % water/THF mixture (Figure 3e).

The bulky donor group in BPy-*p*3C led to facile D–A rotation in solution. As a result, we observed the twisted molecular geometry and a lower PLQY of 3.6% as compared to rigid BPy-*p*TC (PLQY = 12.7%) in THF solution. In contrast to the THF solution, BPy-*p*3C showed enhanced PLQY from 3.6% to 9.8% in an aggregated state, while BPy-*p*TC showed reduced PLQY from 12.7% in THF solution to 7.0% in the aggregated state. The enhanced emission in the aggregated state of BPy-*p*3C as compared to the THF solution was due to the restricted D–A rotation in the aggregated state [21]. As revealed from the photophysical studies and DFT calculations, BPy-*p*3C has a twisted molecular geometry with a stronger excited-state ICT than BPy-*p*TC. As a consequence, it facilitates nonradiative deactivation pathways in solution due to molecular vibrations and facile D–A rotation, as compared to rigid BPy-*p*TC (strong ground-state CT interactions render the D–A complex rigid). In comparison, due to the ground-state D–A communication on the basis of a +R effect, the weak excited-state CT interactions and rigid molecular geometry in BPy-*p*TC led to the regain of fluorescence in the aggregated state [30]. However, the fluorescence intensity of aggregates of BPy-*p*TC did not reach beyond the native THF solution. Another factor that may have been responsible for this were the π–π-stacking interactions in the aggregated or solid phase of BPy-*p*TC as compared to twisted BPy-*p*3C [36].

**Figure 4:**
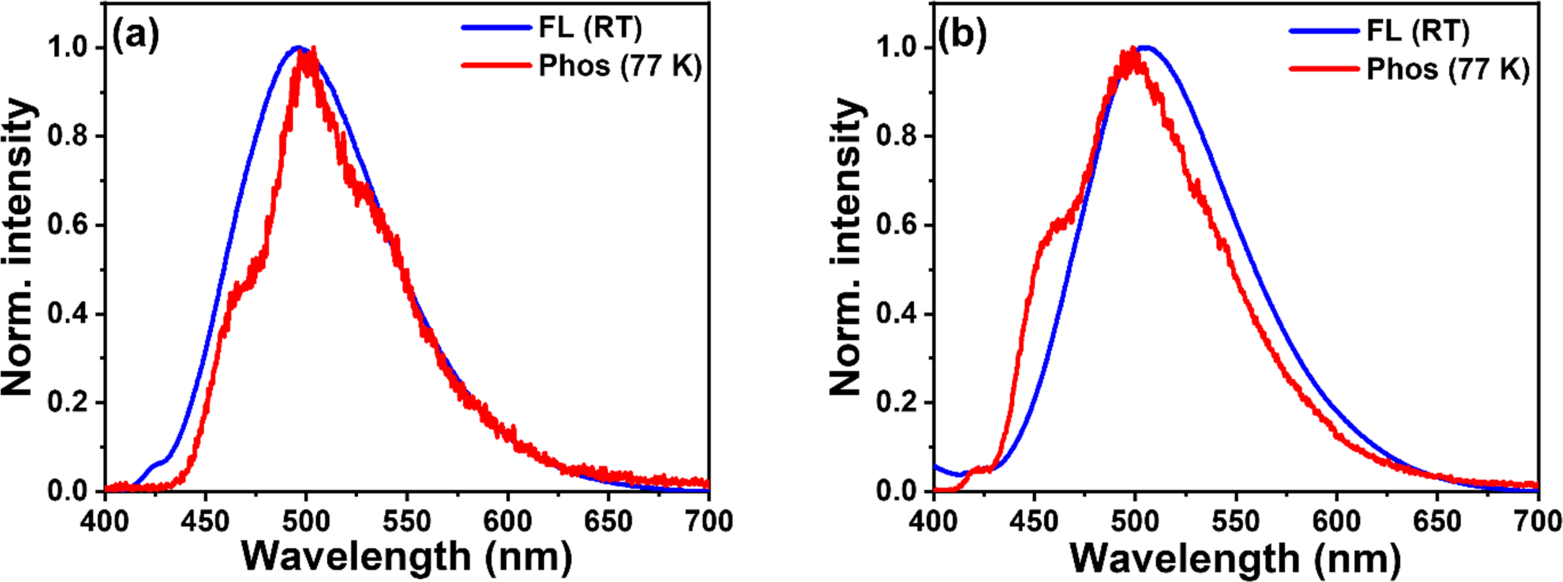
Normalized fluorescence at room temperature and phosphorescence spectra at 77 K (λ_ex_ = 375 nm, 10 µM) of (a) BPy-*p*TC and (b) BPy-*p*3C aggregates in 90 vol % water/THF mixture.

To determine the effect of aggregation on the spin-flipping process (indicated by *k*_RISC_) from the low-lying triplet state T_1_ to the singlet excited state S_1_, we recorded the fluorescence and phosphorescence spectra of aggregates (90 vol % water/THF) at room temperature and 77 K, respectively (Figure 4). The structural phosphorescence band was obtained from the aggregates in 90 vol % water/THF mixtures (Figure 4). Additionally, the phosphorescence spectra of both compounds were recorded in THF at 77 K (Figure S3 in Supporting Information File 1). The retention of the structural phosphorescence band in THF indicates its origin from the locally excited triplet state ^3^LE. The Δ*E*_ST_ values calculated based on the onset of the fluorescence and the phosphorescence spectra of BPy-*p*TC and BPy-*p*3C in 90 vol % water/THF were 0.06 and −0.047 eV, respectively (Figure 4). The Δ*E*_ST_ values were lower in the aggregated state than in solution for both compounds (Δ*E*_ST_ in toluene for BPy-*p*TC and BPy-*p*3C = 0.121 and 0.047 eV, respectively), which indicated aggregation-induced RISC boosting. BPy-*p*3C showed a negative Δ*E*_ST_ value because the phosphorescence emission came from the locally excited triplet states (^3^LE-structured emission), not from the ^3^CT state [37]. This low Δ*E*_ST_ value facilitates fast spin-flipping in the aggregated state. Consequently, a fast *k*_RISC_ rate (0.74·10^5^ s^−1^ for BPy-*p*TC and 2.06·10^5^ s^−1^ for BPy-*p*3C) was obtained in the aggregated state (Table 2). Interestingly, a higher reduction of Δ*E*_ST_ as compared to BPy-*p*TC was obtained for the aggregates of BPy-*p*3C. This could have been due to the locking of the twisted molecular geometry in the aggregated state and more dipolar excited states in the highly polar water/THF mixtures.

**Table 2:** Photophysical properties of BPy-*p*TC and BPy-*p*3C aggregates.

compound	sample	λ_em_^a^ (nm)	Δ*E*_ST_^a^ (eV)	τ^PF^ (ns)	τ^DF^ (µs)	Φ_total_ (%)	Φ_PF_ (%)	Φ_DF_ (%)	*k*_r_ (10^7^ s^−1^)	*k*_ISC_ (10^7^ s^−1^)	*k*_RISC_ (10^5^ s^−1^)	*k*_nr, S_ (10^7^ s^−1^)

BPy-*p*TC	90 vol % water/THF	496	0.06	9.0	19.1	7.0	4.8	2.2	0.5	0.36	0.74	6.9
BPy-*p*3C	90 vol % water/THF	505	−0.047	19.2	6.8	9.8	6.8	3.3	0.4	0.18	2.06	3.2

^a^Measured in 90 vol % water/THF mixture.

**Figure 5:**
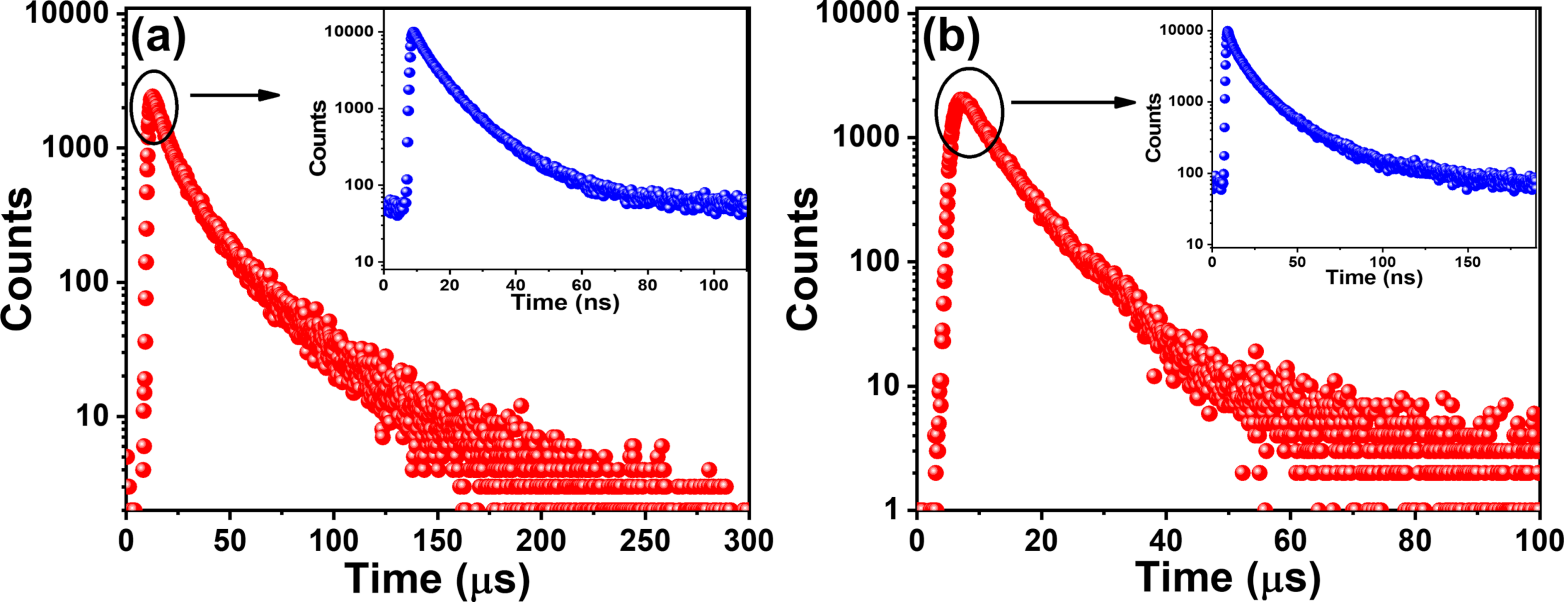
Transient photoluminescence decay (λ_ex_ = 375 nm, 20 µM) of (a) BPy-*p*TC and (b) BPy-*p*3C aggregates in 90 vol % water/THF mixture.

Further, the PF and DF lifetimes of the aggregates of BPy-*p*TC and BPy-*p*3C were measured in degassed solutions (Figure 5 and Table 2). We found an over 1.5- and 3-fold enhancement of the PF and DF lifetimes, respectively, for BPy-*p*TC in the aggregated state as compared to the THF solutions (Figure 5a and Table 2). However, only the PF lifetime was enhanced in BPy-*p*3C (3.5-fold), and no significant change was obtained in the DF lifetime of the aggregates (Figure 5b and Table 2). Therefore, it is evident that the aggregation helped to suppress the fluorescence quenching as well as to boost the DF by reducing the Δ*E*_ST_ in BPy-*p*TC. At the same time, the significant enhancement of the PF lifetime was due to the molecular rigidification and restriction of D–A rotation in BPy-*p*3C aggregates. Thus, a judicious choice of D–A molecular architecture is necessary to tune the solid- or aggregated-state optical properties for triplet harvesting.

### Fluorescence switching

**Figure 6:**
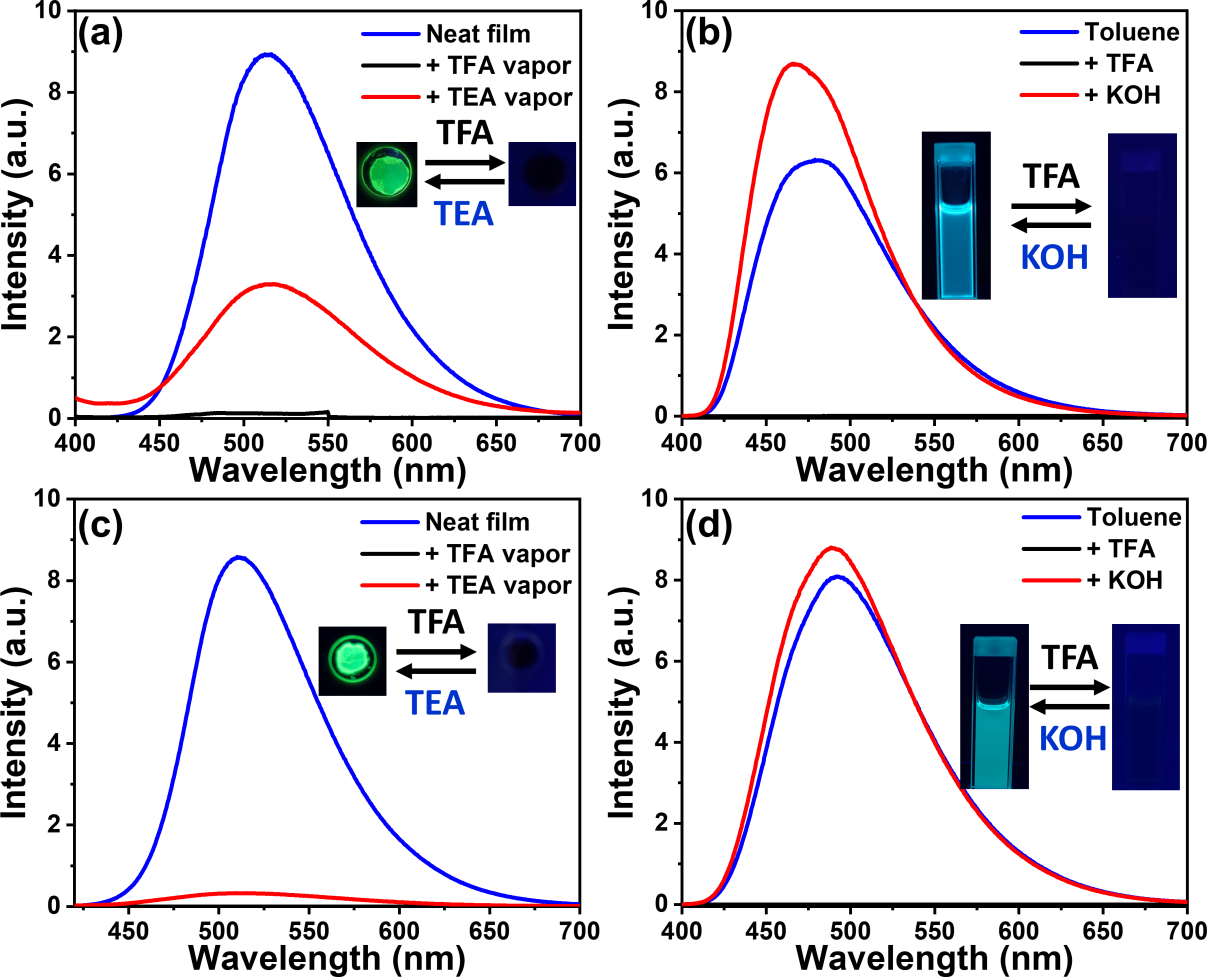
Fluorescence switching by acid and base fumes exposure: Emission spectra (λ_ex_ = 375 nm) of (a) BPy-*p*TC and (c) BPy-*p*3C in neat film upon exposure to TFA and TEA vapor at room temperature. Fluorescence switching by addition of TFA and KOH to (b) BPy-*p*TC and (d) BPy-*p*3C in toluene solution.

The lone-pair electrons of heteroatoms, such as oxygen and nitrogen, are susceptible to acidic protons [22]. BPy-*p*TC and BPy-*p*3C exhibit lone-pair electrons at the pyridinyl nitrogen atom and show prominent emission in the solid and aggregated states. We therefore demonstrated the switching of fluorescence using acid and base vapors in neat film (Figure 6a and Figure 6c). Both emitters exhibited switching of the fluorescence responses upon exposure to acidic and basic fumes on neat film (Figure 6a and Figure 6c). The intense green emission of the BPy-*p*TC film turned dark upon exposure to trifluoroacetic acid (TFA) vapor for 4 s (Figure 6a). The green fluorescence reappeared after neutralization with triethylamine (TEA) vapor for 6 s (Figure 6a). The reversible color switching was upheld with fatigue resistance for multiple cycles. Similarly, the film of BPy-*p*3C showed reversible changes of the fluorescence from green to dark upon successive exposure to TFA and TEA vapors (Figure 6c).

To understand the effect of an acidic solution on the optical properties, we carried out the same experiment in toluene solution (Figure 6b and Figure 6d). The protonation of the pyridinyl nitrogen atom upon addition of TFA quenched the fluorescence of both compounds (Figure 6b and Figure 6d). In turn, neutralization of the toluene solution by TEA or KOH addition led to the regain of fluorescence (Figure 6b and Figure 6d). Further, the Stern–Volmer plots were analyzed for both compounds to obtain the quenching and recovery constants (Figures S4 and S5 in Supporting Information File 1). The linear fitted Stern–Volmer plots for quenching and recovery were obtained for both compounds upon addition of acid and base. Thereby, the protonation–deprotonation events were confirmed using absorption spectroscopy (Figure S6, Supporting Information File 1). The new peak at λ_abs_ = 425 nm in the absorption spectrum of BPy-*p*TC upon adding TFA, indicated the protonation of the pyridinyl nitrogen atom (Figure S6a, Supporting Information File 1). This enhanced the electron-deficient character of the BPy acceptor core and the ground-state communication between the donor and acceptor units. As a result, the red-shifted CT absorption band in the acidic medium was obtained (Figure S6a in Supporting Information File 1). The absorption spectra upon addition of TEA again matched with the absorption spectra in toluene (Figure S6a, Supporting Information File 1).

**Figure 7:**
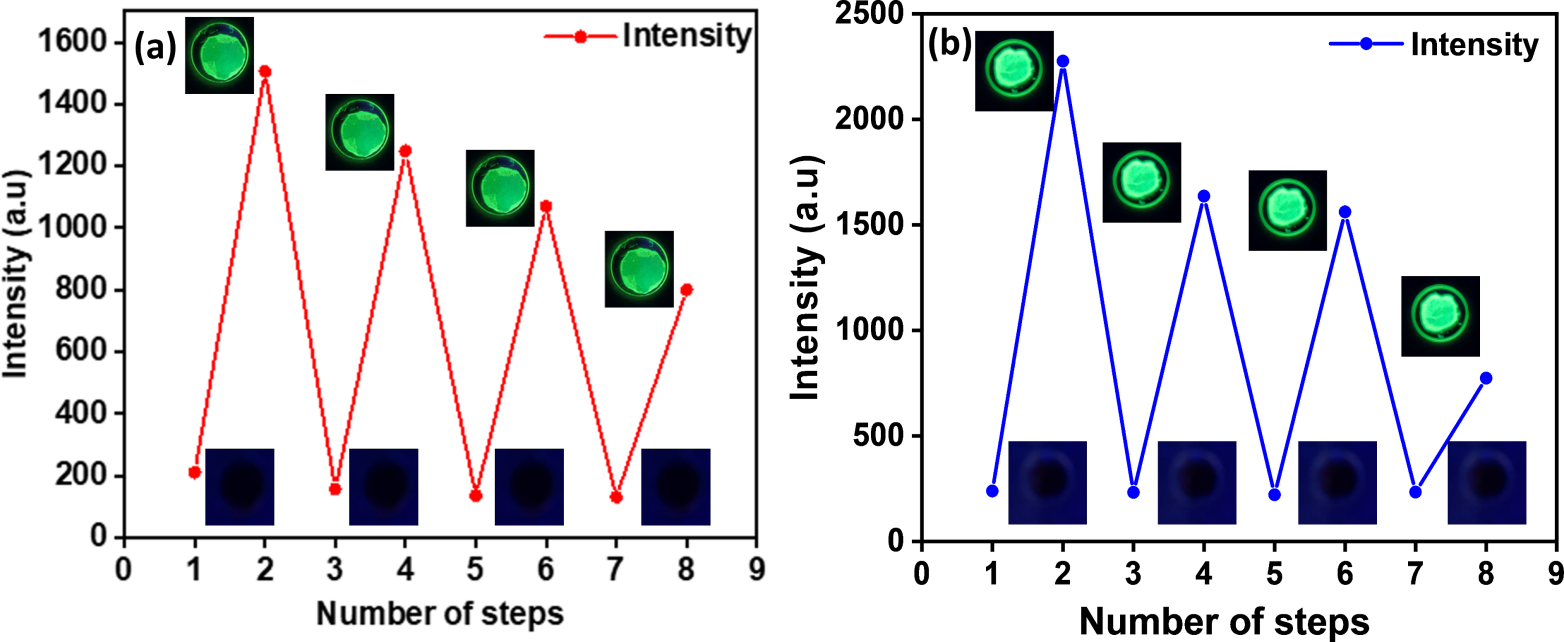
Fluorescence intensity vs number of exposures for (a) BPy-p3C and (b) BPy-pTC thin films upon exposure to TFA and TEA vapors.

Further, TDDFT calculations for a protonated pyridinyl nitrogen atom (N–H) and carbonyl oxygen atom (CO–H) were performed in comparison to neutral BPy-*p*TC to determine the protonation site (Figure S7 in Supporting Information File 1). The computed absorption band of BPy-*p*TC and protonated pyridinyl resembled the change observed experimentally (Figures S6 and S7 in Supporting Information File 1). Additionally, the ^1^H NMR study upon the addition of TFA in BPy-*p*TC suggested protonation of the pyridinyl nitrogen atom (Figure S8, Supporting Information File 1). Thus, the facile protonation of the pyridinyl nitrogen atom affected the optical properties of both compounds. The reversible change of absorption and emission by successive addition of acid and base in solution as well as in the solid state indicated that both compounds are suitable for acid sensing in solution and in the solid phase. Reversible fluorescence for thin emitter films was observed in the presence of TFA and TEA. Essentially, quenching occurs in the presence of acid and regaining of the fluorescence intensity happens under basic conditions (Figure 7).

## Conclusion

In summary, we designed and synthesized two D–A-based TADF emitters, BPy-*p*TC and BPy-*p*3C. Both emitters showed excited-state ICT characteristics, leading to positive solvatochromism in solution. The Lippert–Mataga plot and DFT calculations indicated that BPy-*p*3C had more excited-state CT properties than BPy-*p*TC due to the bulkier donor group. Both compounds displayed strong emission in the aggregated state in a highly polar medium (80–90 vol % water/THF mixtures). As compared to the native solution, BPy-*p*3C showed AIEE. Moreover, as compared to the solution, Δ*E*_ST_ was reduced in the aggregated form. Consequently, a fast RISC rate was obtained for the aggregates of both compounds. Further, these two TADF emitters were used to demonstrate solid-state fluorescence switching upon exposure to TFA and TEA vapors. The current study helps to understand the enhancement of DF in the aggregated state, which is important for the fabrication of efficient OLED devices and reversible switching of fluorescence by acid–base exposure.

## Supporting Information

File 1 General information, synthesis and characterization data including NMR spectra, computational details, UV–vis data, Lippert–Mataga plot, and acid–base switching.
