# Supplementary material for: Thermally activated delayed fluorescence (TADF) emitters: sensing and boosting spin-flipping by aggregation
Source: Beilstein J Org Chem. 2022 Sep 8;18:1177–87. doi: 10.3762/bjoc.18.122 (PMC9475190; doi:10.3762/bjoc.18.122)
Supplement: File 1 — General information, synthesis and characterization data including NMR spectra, computational details, UV–vis data, Lippert–Mataga plot, and acid–base switching. [file Beilstein_J_Org_Chem-18-1177-s001.pdf]

BEILSTEIN JOURNAL OF ORGANIC CHEMISTRY

Supporting Information

for

Thermally activated delayed fluorescence (TADF) emitters: sensing and boosting spin-flipping by aggregation

Ashish Kumar Mazumdar, Gyana Prakash Nanda, Nisha Yadav, Upasana Deori, Upasha Acharyya, Bahadur Sk and Pachaiyappan Rajamalli

Beilstein J. Org. Chem. **2022,** *18,* 1177–1187. doi:10.3762/bjoc.18.122

General information, synthesis and characterization data including NMR spectra, computational details, UV–vis data, Lippert–Mataga plot, and acid–base switching

License and Terms: This is a supporting information file under the terms of the Creative Commons Attribution License (https://creativecommons.org/licenses/by/4.0). Please note that the reuse, redistribution and reproduction in particular requires that the author(s) and source are credited and that individual graphics may be subject to special legal provisions.
The license is subject to the *Beilstein Journal of Organic Chemistry* terms and conditions: (https://www.beilstein-journals.org/bjoc/terms)

Table of contents

1. Materials and methods S1

1.1 Chemicals: S1

1.2 Instrumentation: S1

2. Synthesis and characterizations S2

2.1 Synthesis of (4-(3,6-di-*tert*-butyl-9*H*-carbazol-9-yl)phenyl)(pyridin-4-yl)methanone (BPy-*p*TC) [1] S2

2.2 Synthesis of (4-(9'*H*-[9,3':6',9''-tercarbazol]-9'-yl)phenyl)(pyridin-4-yl)methanone (BPy-*p*3C) S3

3. Computational calculations S4

4. Spectroscopic characterizations S6

4.1 Spectroscopic data: S6

4.2 Lippert–Mataga equation: S6

4.3 Fluorescence quantum yield measurement: S8

4.4 Time-resolved spectroscopy: S8

5. Fluorescence switching S9

6. References S13

7. NMR spectra S14

1. Materials and methods

1.1 Chemicals:

All chemicals were used as received unless otherwise stated. The chemicals were received from Sigma-Aldrich, Merck India, and Alfa aesar.

1.2 Instrumentation:

The ^{1}H and ^{13}C NMR spectra were recorded by using a Bruker Avance 400 spectrometer. The mass spectra were recorded by electrospray ionization (ESI) method on a Q-TOF Micro with lock spray source. The UV–vis absorption spectra were taken on a Jasco V-730 spectrophotometer. Fluorescence and phosphorescence spectra were recorded on a Hitachi F-7100 spectrophotometer. Transient photoluminescence decay measurements were recorded on FLS-980 EDINBURGH spectrometer.

2. Synthesis and characterizations

2.1 Synthesis of (4-(3,6-di-*tert*-butyl-9*H*-carbazol-9-yl)phenyl)(pyridin-4-yl)methanone (BPy-*p*TC) [1]

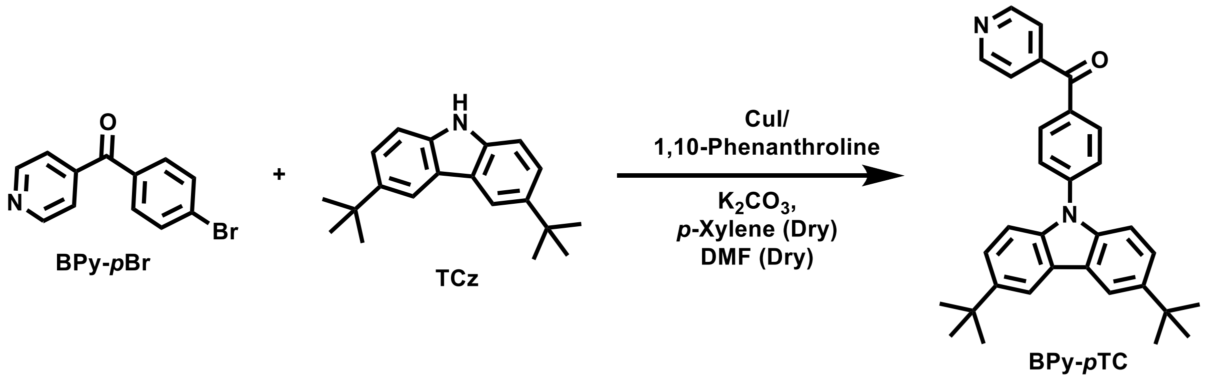

Scheme S1: Synthesis scheme of (4-(3,6-di-*tert*-butyl-9*H*-carbazol-9-yl)phenyl)(pyridin-4-yl)methanone (BPy-*p*TC).

A mixture of 4-bromobenzoylpyridine (0.5 g, 1.9 mmol), *tert*-butyl carbazole (0.58 g, 2 mmol), potassium carbonate (0.526 g, 3.8 mmol), 1,10-phenanthroline (0.075 g, 0.38 mmol), and copper iodide (0.073 g, 0.38 mmol) in dry DMF (5 mL) and xylene (20 mL) mixture was placed in a 50 mL sealed tube under nitrogen environment. The reaction mixture was stirred at a temperature of 165 °C for 30 hours [Scheme S1]. After completion of the reaction, the mixture was passed through celite filtration, the extract was dried using magnesium sulfate, and then the solvent was removed under reduced pressure. The dried residue was then purified by silica gel column chromatography using a 6% ethyl acetate/*n*-hexane mixture as eluent to give the desired white colour compound (yield = 80%). ^{1}H NMR (500 MHz, $CDCl_3$) δ 8.90 – 8.87 (m, 1H), 8.15 (m, 1H), 8.10 – 8.05 (m, 1H), 7.80 – 7.76 (m, 1H), 7.73 – 7.69 (m, 1H), 7.49 (m, 2H), 1.48 (s, 9H). ^{13}C NMR (126 MHz, $CDCl_3$) δ 193.92, 150.18, 144.95, 144.07, 143.52, 138.42, 133.44, 132.11, 125.93, 124.19, 124.06, 123.11, 116.61, 109.38, 34.89, 32.17, 32.05, 27.01. HRMS (ESI-QTOF) m/z: $[M]^+$ calculated for $C_{32}H_{32}N_2O$ 461.2593, found 461.2587.

2.2 Synthesis of (4-(9'*H*-[9,3':6',9''-tercarbazol]-9'-yl)phenyl)(pyridin-4-yl)methanone (BPy-*p*3C)

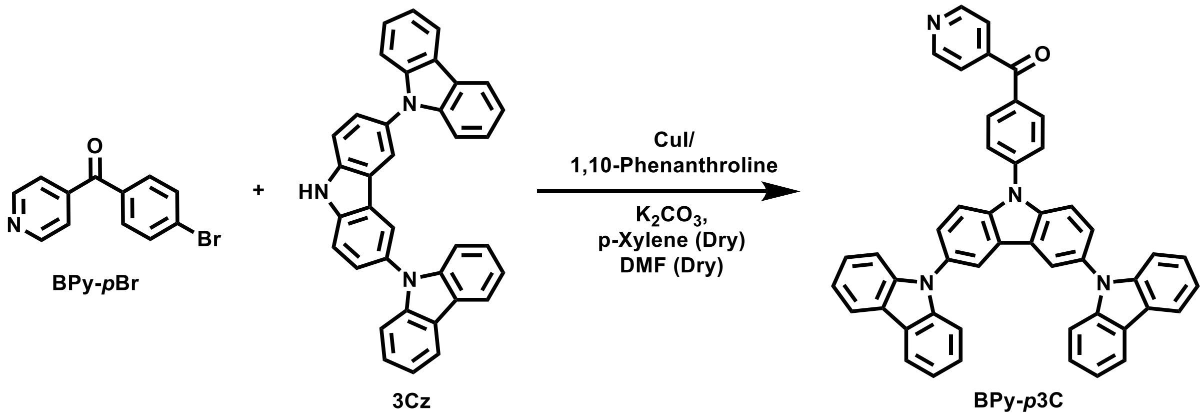

Scheme S2: Synthesis scheme of (4-(9'*H*-[9,3':6',9''-tercarbazol]-9'-yl)phenyl)(pyridin-4-yl)methanone (BPy-*p*3C).

Synthesis of BPy-p3C, followed the similar procedure as described for BPy-pTC. A mixture of 4-bromobenzoylpyridine (0.5 g, 1.9 mmol), tricarbazole (1.04 g, 2 mmol), potassium carbonate (0.526 g, 3.8 mmol), 1,10-phenanthroline (0.075 g, 0.38 mmol), and copper iodide (0.073 g, 0.38 mmol) in dry DMF (5 mL) and xylene (20 mL) mixture was placed in a 50 mL sealed tube under nitrogen environment, and the reaction mixture was stirred at a temperature of 165 °C for 30 hours [Scheme S2]. After completion of the reaction, the mixture was passed through celite filtration, the extract was dried using magnesium sulfate, and then the solvent was removed under reduced pressure. The dried residue was then purified by silica gel column chromatography using a 6% ethyl acetate/*n*-hexane mixture as eluent to give the desired white colour compound (yield = 80%). ^{1}H NMR (500 MHz, $CDCl_3$) δ 8.94 – 8.90 (m, 1H), 8.33 (m, 1H), 8.19 (dd, J = 8.1, 6.6 Hz, 3H), 7.94 (d, J = 8.4 Hz, 1H), 7.79 (d, J = 8.7 Hz, 1H), 7.75 – 7.72 (m, 1H), 7.68 (dd, J = 8.7, 2.0 Hz, 1H), 7.43 (d, J = 4.0 Hz, 4H), 7.31 (dt, J = 8.1, 4.1 Hz, 2H). ^{13}C NMR (126 MHz, $CDCl_3$) δ 193.91, 150.47, 144.39, 142.17, 141.76, 139.98, 134.95, 132.41, 131.32, 126.83, 126.68, 126.32, 126.10, 125.86, 124.76, 123.36, 123.06, 120.51, 120.06, 119.99, 111.43, 109.74. HRMS (ESI-QTOF) m/z: $[M]^{+}$ calculated for $C_{32}H_{32}N_2O$ 678.2407, found 678.2402.

3. Computational calculations

A time-dependent density functional theory (TDDFT) investigation for BPy-*p*TC and BPy-*p*3C was performed using the Gaussian 16 program package in the delta-cluster of SERC facility @IISc. The ground state molecular geometries of D–A compounds were optimized employing density functional theory (DFT) B3LYP hybrid functional and 6-31G(d,p) basis set in Gaussian 16 software [2]. The TDDFT calculations with the same functionality and the basis set were employed to obtain the excited-state structure. GaussView 5.0 and Chemcraft (version 1.8) were used to analyze the molecular orbitals. The iso values ±0.03 were used for the HOMO–LUMO orbital picture. The spatial distributions of the highest occupied molecular orbital (HOMO) and the lowest unoccupied molecular orbital (LUMO) of BPy-*p*TC and BPy-*p*3C in the ground were analyzed (Figure S1).

Table S1: The salient features of the most probable electronic transitions with oscillator strengths of BPy-*p*TC and BPy-*p*3C were obtained from the TDDFT calculations at the B3LYP/6-31G(d,p) level of theory.

**Compound**	λ_{ex}	***f***	**Transitions**
**BPy-*p*TC**	436	0.2132	HOMO to LUMO (99%)
	314	0.0299	HOMO to L+2 (93%)
	311	0.0761	HOMO to L+1 (87%)
	302	0.0167	H-4 to LUMO (55%), H-3 to LUMO (12%), HOMO to L+3 (11%)
	300	0.0136	H-4 to LUMO (11%), HOMO to L+3 (57%)
**BPy-*p*3C**	481	0.0726	HOMO to LUMO (99%)
	371	0.168	H-4 to LUMO (96%)
	369	0.0189	HOMO to L+1 (97%)
	368	0.0242	H-1 to L+1 (97%)
	358	0.0316	HOMO to L+2 (96%)

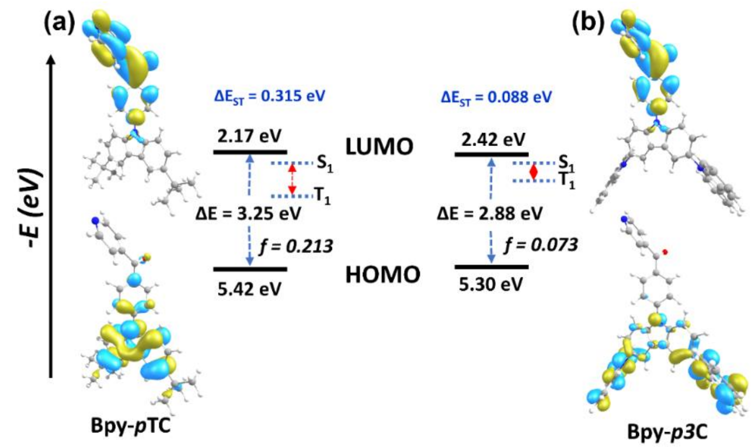

Figure S1: DFT calculated electron density distributions of the highest occupied molecular orbital (HOMO) and the lowest unoccupied molecular orbital (LUMO) of (a) 3BPy-*p*TC and (b) 3BPy-*p*3C at the B3LYP/6-31G(d,p) level of theory. (Isovalue = ±0.03).

Table S2: HOMO distribution and energy values of TCz and 3Cz donor were estimated by DFT at the B3LYP/6-31G(d,p) level.

Donor	E_{HOMO} (-eV)	HOMO
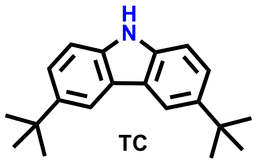	5.23	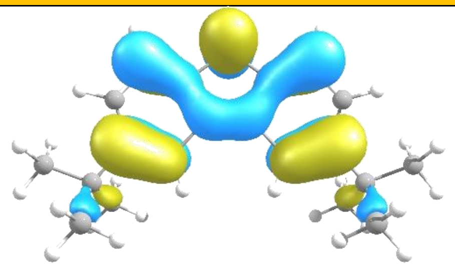
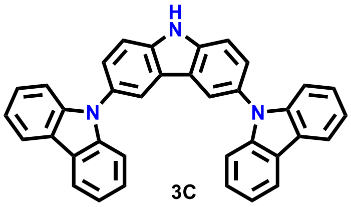	5.21	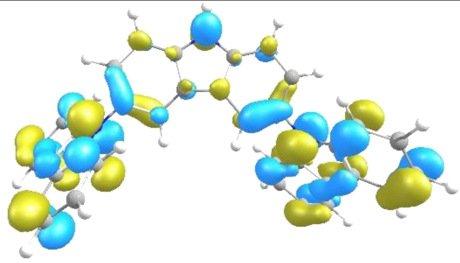

4. Spectroscopic characterizations

4.1 Spectroscopic data:

Table S3: Spectroscopic data table of BPy-*p*TC

Solvent	Orientation polarizability (Δf)	λ_{abs} (nm)	λ_{em} (nm)	Stokes shift (nm)	Δυ (cm^{-1})
TL	0.014	383	477	**94**	5145
DIO	0.025	384	484	**100**	5380
THF	0.210	381	497	**116**	6126
DCM	0.217	383	545	**162**	7761

Table S4: Spectroscopic data table of BPy-*p*3C

Solvent	Orientation polarizability (Δf)	λ_{abs} (nm)	λ_{em} (nm)	Stokes shift (nm)	Δυ (cm^{-1})
TL	0.014	368	487	**119**	6640
DIO	0.025	376	510	**134**	6988
THF	0.210	365	530	**165**	8530
DCM	0.217	368	582	**214**	9991

4.2 Lippert–Mataga equation:

The Lippert–Mataga (L–M) theory describes the solvent dependency of spectral shifts as given in Equation 1 [3].

$$\Delta\nu = \bar{\nu}_a - \bar{\nu}_f = \frac{2}{hc}\left(\frac{\varepsilon - 1}{2\varepsilon + 1} - \frac{n^2 - 1}{2n^2 + 1}\right)\frac{(\mu_E - \mu_G)^2}{a^3} + constant \quad .... \ (1)$$

Where: $\bar{\nu}_a = \frac{1}{\lambda_{abs}^{max}}$, $\bar{\nu}_f = \frac{1}{\lambda_{em}^{max}}$ $\quad and \ \Delta f = \left(\frac{\varepsilon-1}{2\varepsilon+1} - \frac{n^2-1}{2n^2+1}\right) \quad .... \ (2)$

Equation 1 shows that the Stokes shift ($\Delta\bar{\nu}$) depends on the dipole moments of the fluorophore in the ground (μ_G) and the excited (μ_E) state, respectively. It also depends on the dielectric constant (ε) and the refractive index (η) of the corresponding solvent. $\bar{\nu}_a$ and $\bar{\nu}_f$ represent the wavenumbers of the absorption and the fluorescence emission, respectively, *h* is the Planck's constant, *c* is the speed of light in vacuum, and *a* is the Onsager radius of the cavity in which the fluorophore resides. Δf is the orientation polarizability of the solvent (Equation 2). Plotting the Stokes shift as a function of the orientation polarizability of the solvents gives the Lippert–Mataga plot (Figure S2).

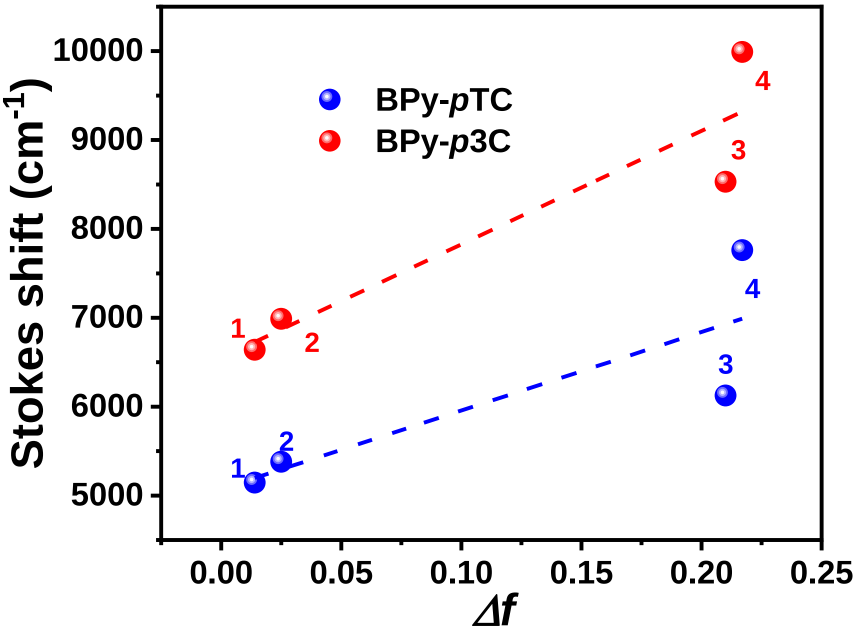

Figure S2: Lippert–Mataga plot depicting Stokes shift ($\Delta\overline{\nu}$) versus the solvent orientation polarizability (Δ*f*) of BPy-*p*TC and BPy-*p*3C. The numbers refer to the solvents: (1) toluene, (2) dioxane, (3) tetrahydrofuran, and (4) dichloromethane. The dashed line represents the best linear fit to the data points.

The change of the dipole moment (Δμ) can be estimated using the equation (1) from the slope obtained from the L–M plot as following

$$(\mu_E - \mu_G)^2 = \frac{Slope * (\mathrm{h}ca^3)}{2} \ldots\ (3)$$

Δ μ($\mu_E - \mu_G$) is the change of dipole moment from the ground to an excited state.

Table S5: Fitting parameters of Lippert–Mataga plots and calculated Onsager radius (a), Δμ ($\mu_E - \mu_G$) refers to the change in dipole moment. (R^2: regression coefficient)

Compound	Intercept	Slope (cm^{-1})	R^2	a (Å)#	(μ_E - μ_G) D
BPy-*p*TC	5074	8831	0.70	8.82	**24**
BPy-*p*3C	6549	12766	0.86	11.35	**43**

#The Onsager radius can be calculated from the optimized geometry structure of the compounds using DFT minimization in Gaussian program (B3LYP functional using 6-31G(d,p) orbital base [4].

4.4 Fluorescence quantum yield measurement:

The photoluminescence quantum yield (PLQY) of BPy-*p*TC and BPy-*p*3C were estimated by comparison with 9,10-diphenylanthracene dye in toluene (Φ_f = 0.95) [5].

$$\Phi_{f,x} = \Phi_{f,s} \times \frac{F_X}{F_S} \times \frac{f_S}{f_X} \times \frac{n_X^2}{n_S^2} \text{....} \quad (4)$$

Where Φ_f is the fluorescence quantum yield, the subscript x denotes sample, and the subscript s refers to the standard dye. F denotes integral fluorescence, n refers to the refractive index of the solvent used in the measurements and f is the absorption factor at the excitation wavelength given by the following equation: $f = 1 - 10^{-\varepsilon(\lambda_{ex})cl} = 1 - 10^{-A(\lambda_{ex})}$, where A is the absorbance, and ε = molar extinction coefficient in L mol^{-1} cm^{-1}.

4.5 Time-resolved spectroscopy:

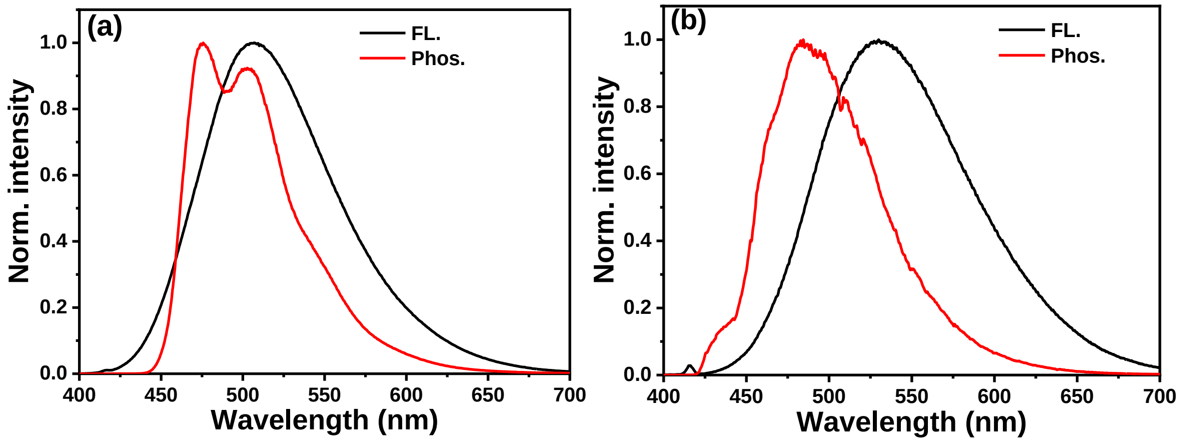

Figure S3: Normalized fluorescence at room temperature and phosphorescence spectra at 77 K (λ_{ex} = 375 nm, 10 µM) of (a) BPy-*p*TC and (b) BPy-*p*3C in THF.

Table S6: Emission decay parameters (λ_{ex}= 375 nm, λ_{em}= 510 nm) of BPy-*p*TC in THF and 90 vol % water/THF fraction at room temperature; the decay times (τ_1, and τ_2), the respective fractional contributions (α_1, and α_2 mentioned in parentheses), the weighted average decay time ($\tau_{avg.}$) and the quality of fitting ($\chi2$) are shown.

Sample	Timescales	τ_1	α_1	τ_2	α_2	$\tau_{avg.}$	$\chi2$
**THF (degassed)**	ns	6.2	100	-	-	-	1.19
	µs	5.8	100	-	-	-	1.15
**90% water-THF**	ns	4.7	46	12.7	54	9.02	1.14
	µs	7.8	46	28.7	54	19.08	1.29

Table S7: Emission decay parameters (λ_{ex}= 375 nm) of BPy-*p*3C in THF and 90 vol % water-THF fraction at room temperature; the decay times (τ_1, and τ_2), the respective fractional contributions (α_1, and α_2 mentioned in parentheses), the weighted average decay time ($\tau_{avg.}$) and the quality of fitting ($\chi 2$) are shown.

Sample	Timescales	τ_1	α_1	τ_2	α_2	$\tau_{avg.}$	$\chi 2$
**THF (degassed)**	ns	3.3	99	27.1	1	3.54	1.00
	μs	3.9	42	8.5	58	6.57	1.29
**90% water-THF**	ns	8.2	43	27.5	57	19.20	1.31
	μs	4.2	45	9.0	55	6.84	1.17

5. Fluorescence switching

The Stern–Volmer quenching and recovery constant for the compounds were analyzed. The Stern–Volmer equation is

$$\frac{I_o}{I} = 1 + k_{SV}[Q/R] \qquad \text{eq. 1}$$

Where, I_o is the emission intensity without quencher, I is the emission intensity with quenching/recovering agent, $[Q/R]$ is the concentration of quenching/recovering agent, and k_{SV} is the Stern–Volmer constant. Fluorescence titration experiments were performed by addition of TFA and TEA for both emitters (Figures S4 and S5). Stern–Volmer plots were obtained by plotting I_0/I against acid/base concentration [Q/R], where I_0 is the fluorescence intensity of the emitter when [Q/R] = 0 and I is the intensity at a particular concentration of acid/base [Q/R]. The linear fitting of Stern–Volmer plots was obtained for both quenching and recovery in both cases (Figures S4 and S5). The k_{SV} for quenching and recovery of BPy-pTC is found to 9.7×10^4 and 7.5×10^5, respectively. Whereas, for BPy-p3C 2.8×10^5 and 1.0×10^7 are the quenching and recovery k_{SV} constants, respectively (Figures S4 and S5).

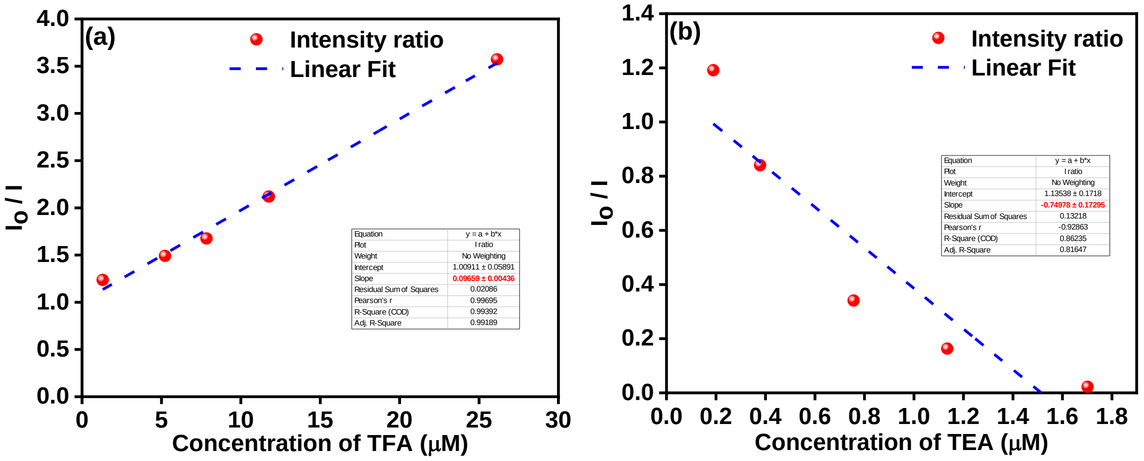

Figure S4: Stern–Volmer plots for BPy-*p*TC of I_0/I against the concentration of (a) trifluoroacetic acid (TFA) and (b) triethylamine (TEA) (I_0 = fluorescence intensity without acid/base); blue dashed lines represent the fit to the respective plot.

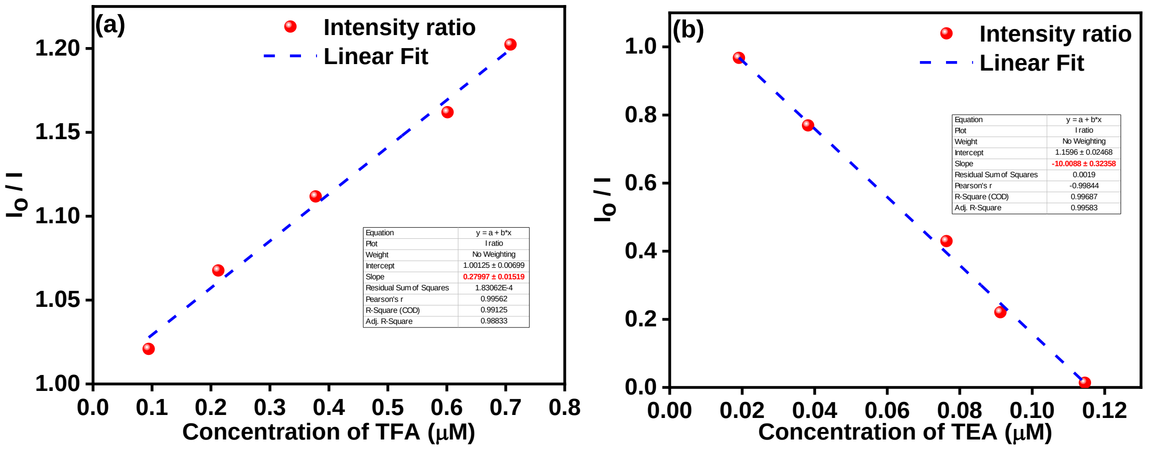

Figure S5: Stern–Volmer plots for BPy-*p*3C of I_0/I against the concentration of (a) trifluoroacetic acid (TFA) and (b) triethylamine (TEA) (I_0 = fluorescence intensity without acid/base); blue dashed lines represent the fit to the respective plot.

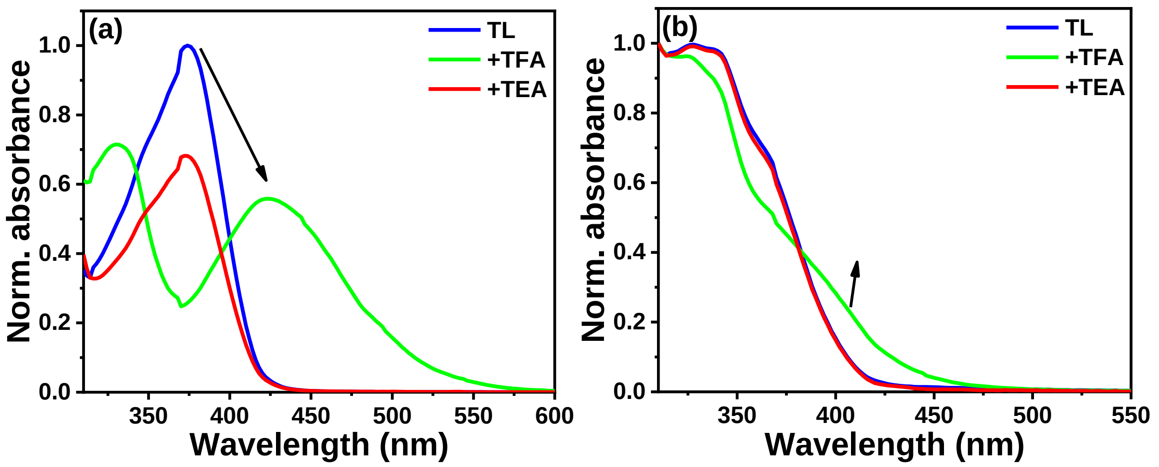

Figure S6: Normalized absorption spectra of (a) BPy-*p*TC and (b) BPy-*p*3C in toluene (TL), addition of 0.1 mL trifluoroacetic acid (TFA) and addition of 0.1 mL triethylamine (TEA) in the same sample vial.

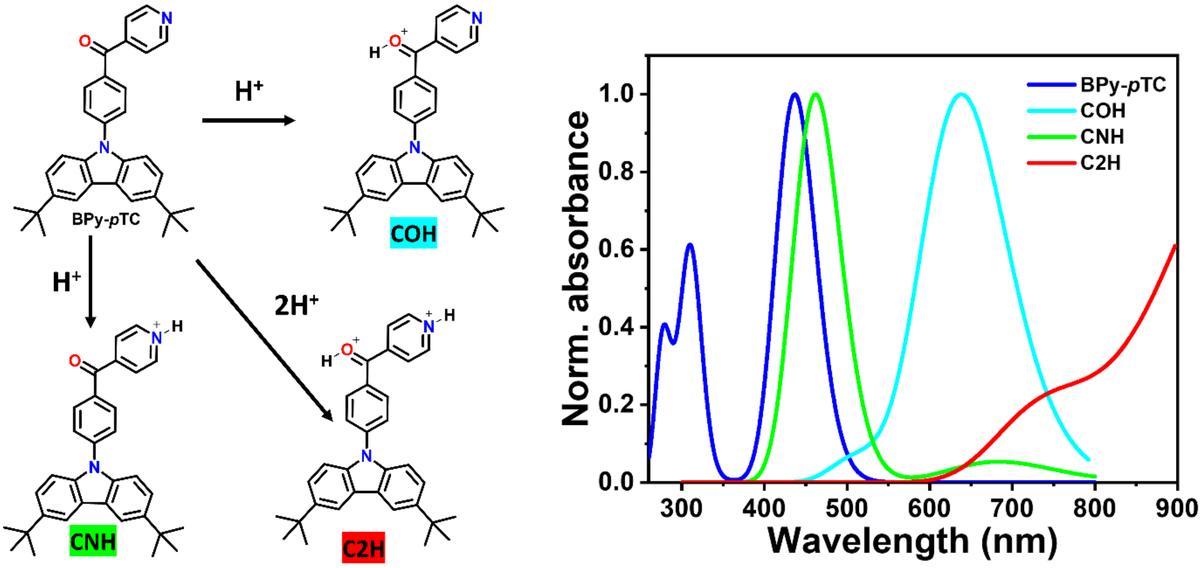

Figure S7: Schematic illustration of proposed molecular structures upon addition of acid in BPy-*p*TC and the corresponding computed absorption spectra of the different protonated forms of BPy-*p*TC.

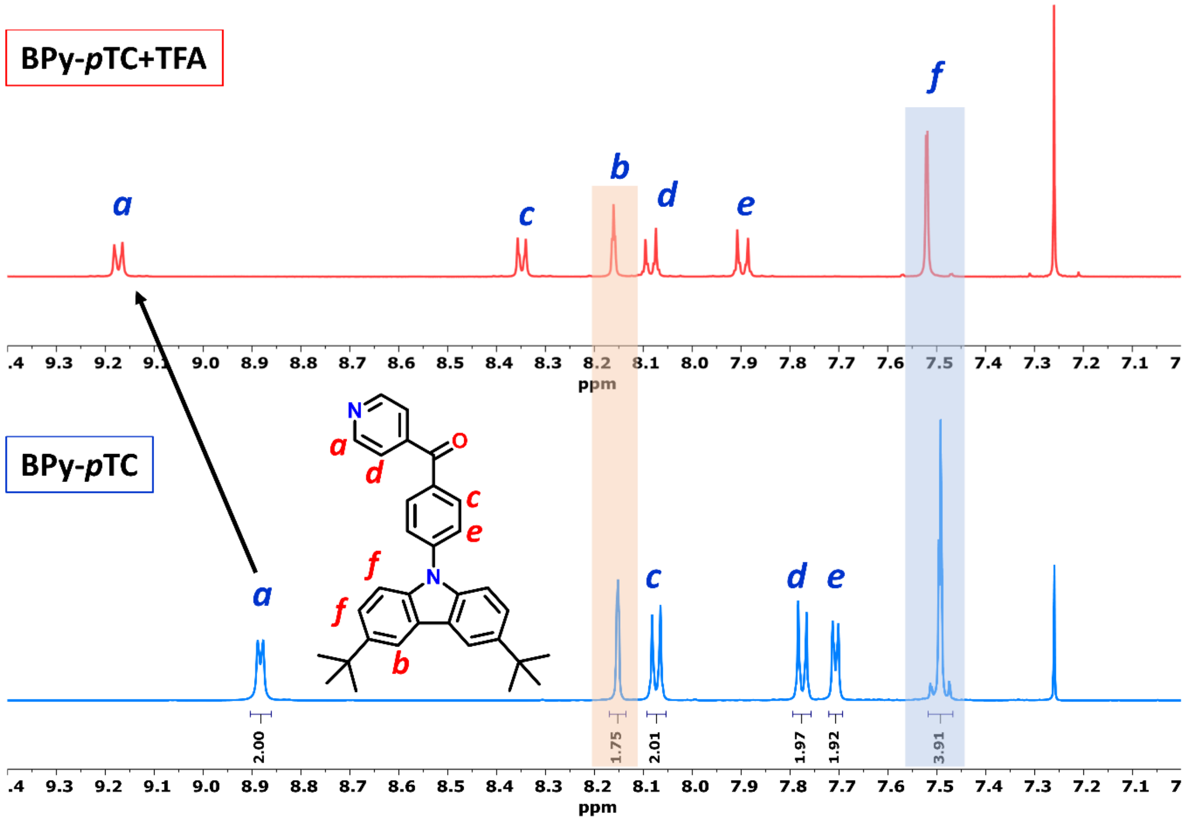

Figure S8: ^{1}H NMR spectra (stacked) of BPy-pTC in $CDCl_3$ before (bottom) and after (top) the addition of TFA.

6. References

1. (a) Rajamalli, P.; Thangaraji, V.; Senthilkumar, N.; Ren-Wu, C.-C.; Lin, H.-W.; Cheng, C.-H. *J. Mater. Chem. C* **2017**, *5*, 2919-2926. DOI: 10.1039/c7tc00457e. (b) Rajamalli, P.; Senthilkumar, N.; Gandeepan, P.; Ren-Wu, C.-C.; Lin, H.-W.; Cheng, C.-H. *ACS Appl. Mater. Interfaces* **2016**, *8*, 27026-27034. DOI: 10.1021/acsami.6b10678.

2. *Gaussian 16*, Revision C.01, Frisch, M. J.; Trucks, G. W.; Schlegel, H. B.; Scuseria, G. E.; Robb, M. A.; Cheeseman, J. R.; Scalmani, G.; Barone, V.; Petersson, G. A.; Nakatsuji, H.; Li, X.; Caricato, M.; Marenich, A. V.; Bloino, J.; Janesko, B. G.; Gomperts, R.; Mennucci, B.; Hratchian, H. P.; Ortiz, J. V.; Izmaylov, A. F.; Sonnenberg, J. L.; Williams-Young, D.; Ding, F.; Lipparini, F.; Egidi, F.; Goings, J.; Peng, B.; Petrone, A.; Henderson, T.; Ranasinghe, D.; Zakrzewski, V. G.; Gao, J.; Rega, N.; Zheng, G.; Liang, W.; Hada, M.; Ehara, M.; Toyota, K.; Fukuda, R.; Hasegawa, J.; Ishida, M.; Nakajima, T.; Honda, Y.; Kitao, O.; Nakai, H.; Vreven, T.; Throssell, K.; Montgomery, J. A., Jr.; Peralta, J. E.; Ogliaro, F.; Bearpark, M. J.; Heyd, J. J.; Brothers, E. N.; Kudin, K. N.; Staroverov, V. N.; Keith, T. A.; Kobayashi, R.; Normand, J.; Raghavachari, K.; Rendell, A. P.; Burant, J. C.; Iyengar, S. S.; Tomasi, J.; Cossi, M.; Millam, J. M.; Klene, M.; Adamo, C.; Cammi, R.; Ochterski, J. W.; Martin, R. L.; Morokuma, K.; Farkas, O.; Foresman, J. B.; Fox, D. J. Gaussian, Inc., Wallingford CT, 2016.

3. (a) Lakowicz, J. R. *Principles of fluorescence spectroscopy*; Springer, 2006. (b) Sk, B.; Khodia, S.; Patra, A. *Chem. Commun.* **2018**, *54*, 1786-1789, DOI: 10.1039/C7CC09261J.

4. Mukherjee, S.; Chattopadhyay, A.; Samanta, A.; Soujanya, T. *J. Phys. Chem.* **1994**, *98*, 2809-2812. DOI: 10.1021/j100062a014.

5. (a) Rurack, K.; Spieles, M. *Anal. Chem.* **2011**, *83*, 1232-1242. DOI: 10.1021/ac101329h. (b) Wurth, C.; Grabolle, M.; Pauli, J.; Spieles, M.; Resch-Genger, U. *Nature protocols* **2013**, *8*, 1535-1550. DOI: 10.1038/nprot.2013.087.

7. NMR spectra

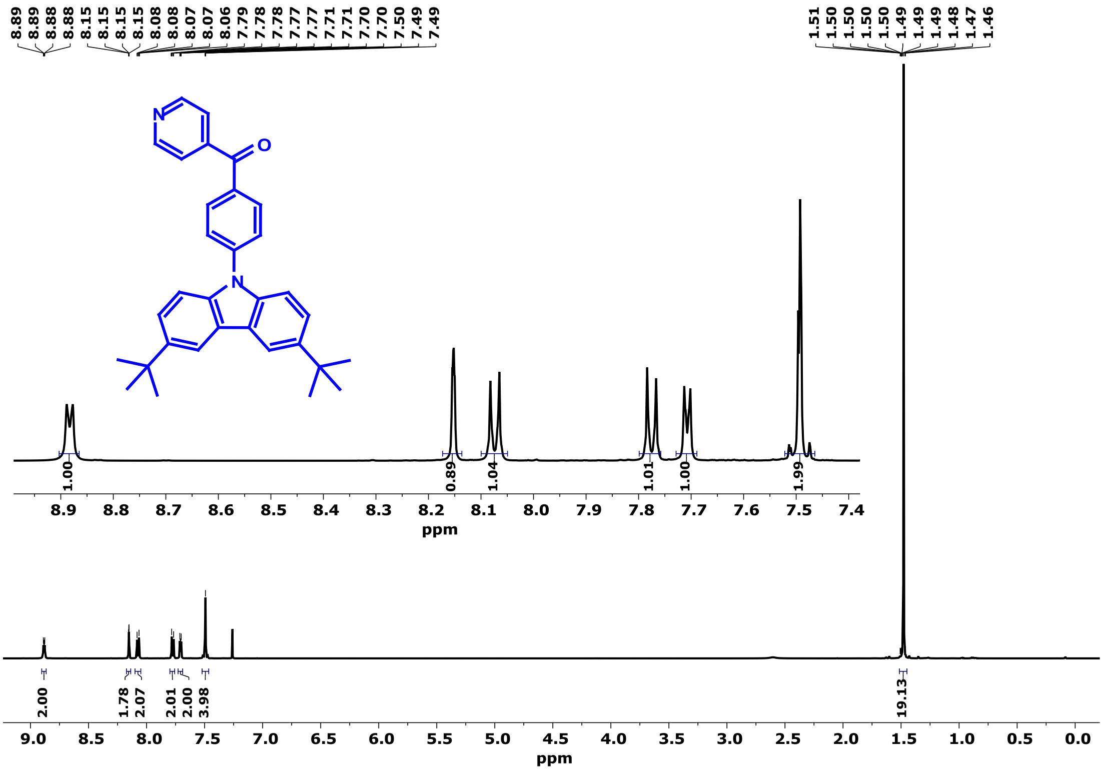

Figure S9: ^{1}H NMR spectrum of (4-(3,6-di-*tert*-butyl-9*H*-carbazol-9-yl)phenyl)(pyridin-4-yl)methanone (**BPy-*p*TC**) in $CDCl_3$.

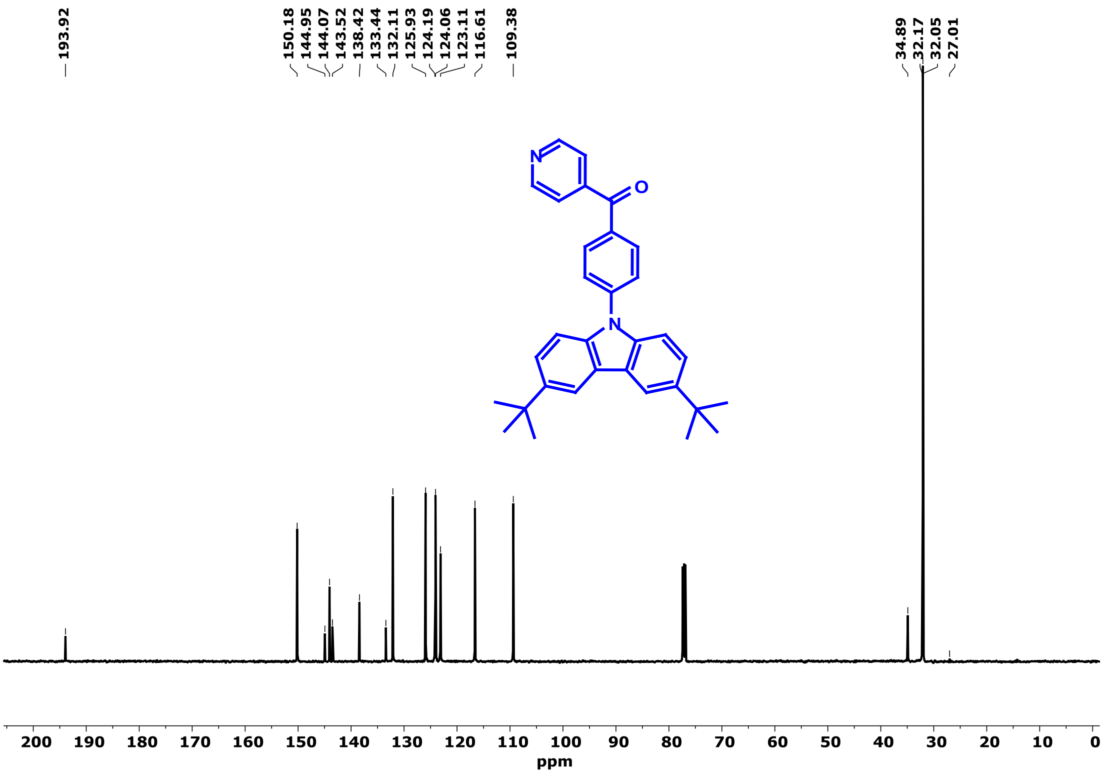

Figure S10: ^{13}C NMR spectrum of (4-(3,6-di-*tert*-butyl-9*H*-carbazol-9-yl)phenyl)(pyridin-4-yl)methanone (**BPy-*p*TC**) in $CDCl_3$.

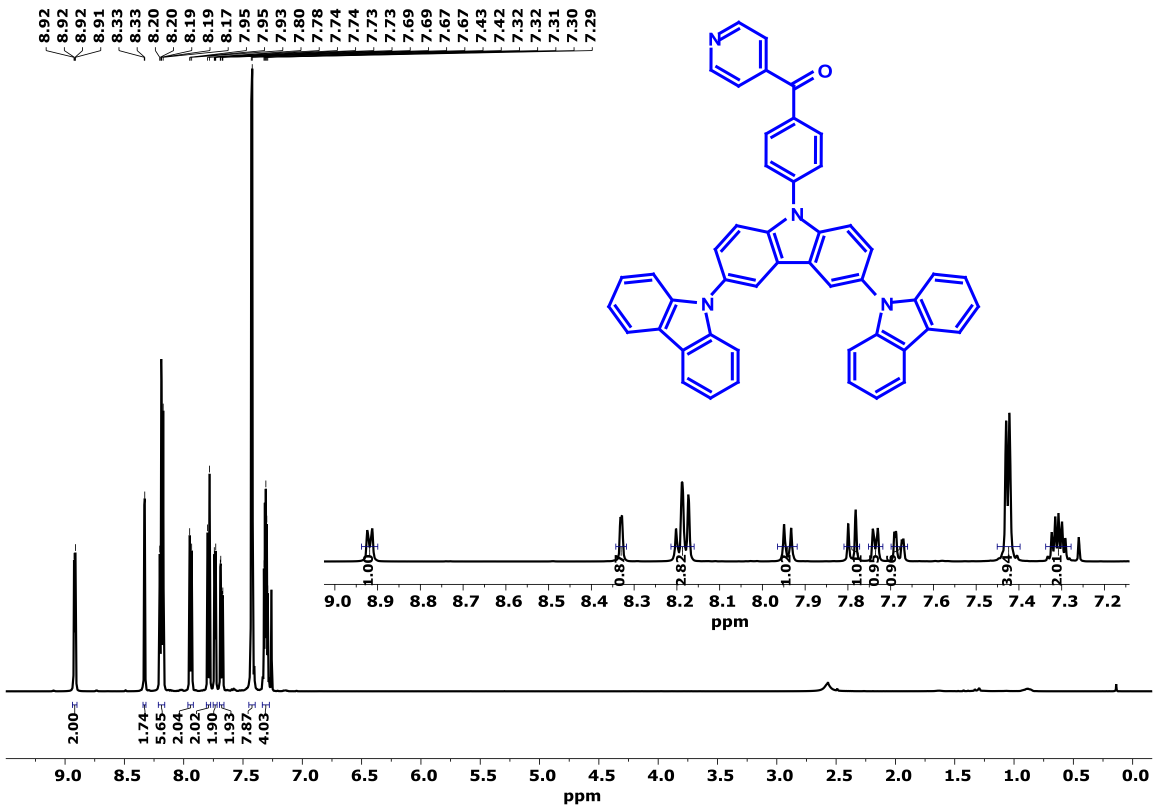

Figure S11: ^{1}H NMR spectrum of (4-(9'*H*-[9,3':6',9''-tercarbazol]-9'-yl)phenyl)(pyridin-4-yl)methanone (**BPy-*p*3C**) in $CDCl_3$.

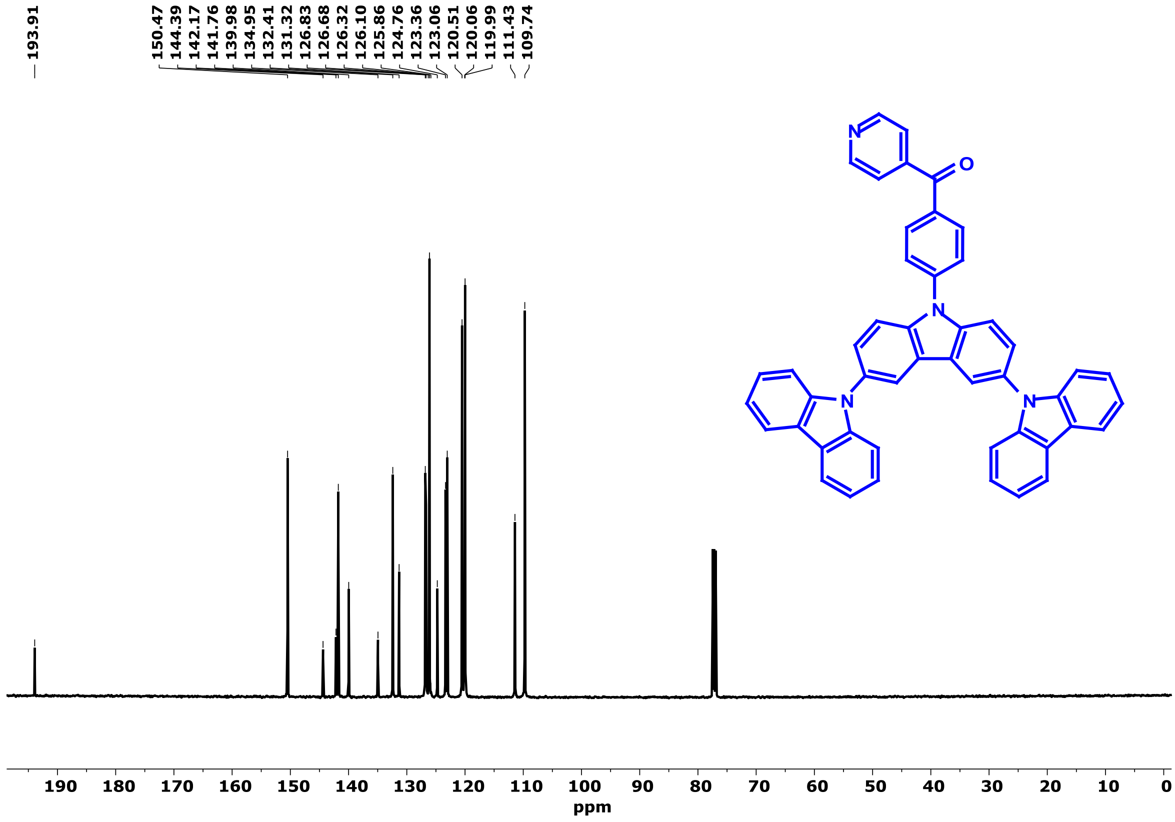

Figure S12: ^{13}C NMR spectrum of (4-(9'*H*-[9,3':6',9''-tercarbazol]-9'-yl)phenyl)(pyridin-4-yl)methanone (**BPy-*p*3C**) in $CDCl_3$.
